# Genetic Traces of Recent Long-Distance Dispersal in a Predominantly Self-Recruiting Coral

**DOI:** 10.1371/journal.pone.0003401

**Published:** 2008-10-14

**Authors:** Madeleine J. H. van Oppen, Adrian Lutz, Glenn De'ath, Lesa Peplow, Stuart Kininmonth

**Affiliations:** Australian Institute of Marine Science, Townsville MC, Queensland, Australia; University of Bristol, United Kingdom

## Abstract

**Background:**

Understanding of the magnitude and direction of the exchange of individuals among geographically separated subpopulations that comprise a metapopulation (connectivity) can lead to an improved ability to forecast how fast coral reef organisms are likely to recover from disturbance events that cause extensive mortality. Reef corals that brood their larvae internally and release mature larvae are believed to show little exchange of larvae over ecological times scales and are therefore expected to recover extremely slowly from large-scale perturbations.

**Methodology/Principal Findings:**

Using analysis of ten DNA microsatellite loci, we show that although Great Barrier Reef (GBR) populations of the brooding coral, *Seriatopora hystrix*, are mostly self-seeded and some populations are highly isolated, a considerable amount of sexual larvae (up to ∼4%) has been exchanged among several reefs 10 s to 100 s km apart over the past few generations. Our results further indicate that *S. hystrix* is capable of producing asexual propagules with similar long-distance dispersal abilities (∼1.4% of the sampled colonies had a multilocus genotype that also occurred at another sampling location), which may aid in recovery from environmental disturbances.

**Conclusions/Significance:**

Patterns of connectivity in this and probably other GBR corals are complex and need to be resolved in greater detail through genetic characterisation of different cohorts and linkage of genetic data with fine-scale hydrodynamic models.

## Introduction

Larval dispersal and reproductive population connectivity (i.e., the dispersal of individuals among subpopulations that survive to reproduce) of most marine populations is poorly understood [1], particularly for reef corals and over recent rather than evolutionary timescales [2], [3]. This limits our ability to evaluate the design and potential benefits of novel conservation and resource management strategies. Knowledge of dispersal distances and pathways over ecological time scales is important as it will provide information regarding the recovery potential of reef coral populations that have suffered mass mortality. Recovery will occur through regrowth of surviving coral colonies and colony fragments, and through new recruitment from local and external sources. However, although some adult coral colonies seem to be able to survive severe disturbances, their presence does not guarantee replenishment because fecundity in addition to adult density determines recruitment densities [4], [5]. As well as reducing adult densities, disturbances may reduce fecundity [6], [7] and may also lower survival of eggs, larvae and juveniles [8], [9]. Recruitment from external sources is therefore likely to be extremely important for recovery after severe but localised environmental perturbations.

The Great Barrier Reef (GBR) is the world's largest reef system (∼350,000 km^2^, of which ∼21,000 km^2^ consists of coral reef) and comprises ∼2,900 separate reefs [10]. Like most other coral reefs in the world it has been affected by both anthropogenic and natural disturbances. Reef corals that brood their larvae internally and release mature larvae are generally believed to show little exchange of larvae over ecological times scales [11]. It is therefore expected that such corals are extremely slow in recovering from large-scale perturbations. The brooding scleractinian (stony) coral, *Seriatopora hystrix* Dana 1846, is a widespread and common species on the GBR [12], and is among the most sensitive species to coral bleaching [13]. Hence, this species is under severe threat from climate change related warming and it is unclear whether damaged populations can be repopulated from external sources.

We follow a genetic approach to obtain an indirect measure of reproductive population connectivity [2], [3], [14] in *S. hystrix* from the central to northern GBR. Previous studies assessing the connectivity of scleractinian corals on the GBR, Australia, have either been conducted over small spatial scales [15], have involved a small number of sampling locations [16], [17], or have used small numbers of loci [18], [19] and/or allozymes rather than DNA markers [20]. Our study is based on a large sample size (1,025 colonies from 22 collection sites) and 10 DNA loci, and as a consequence reveals several new findings with respect to the temporal and spatial scale of connectivity among populations of this coral species on the GBR.

## Results

**Figure 1:**
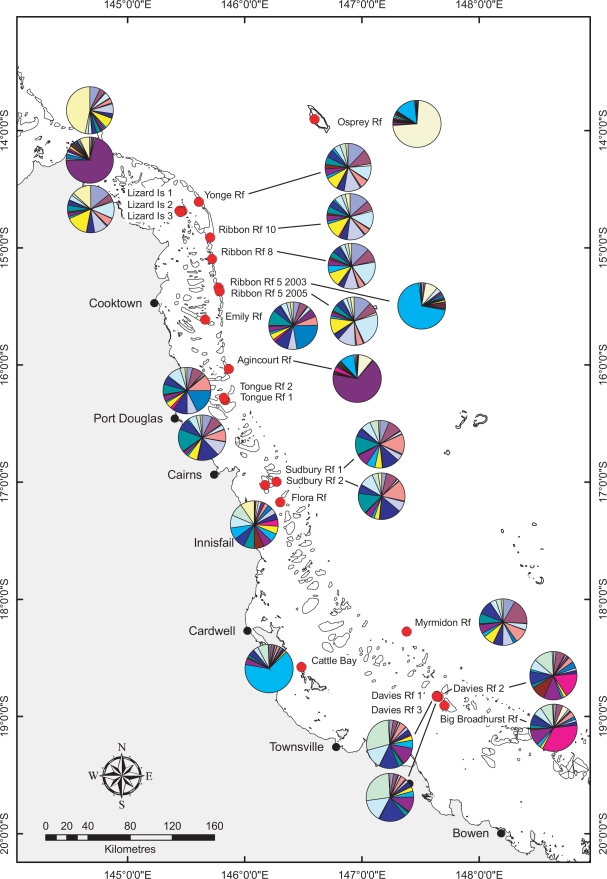
Genetic composition of *S. hystrix* at the 22 sampling sites based on the model-based clustering method implemented in STRUCTURE v2.2 [21].

**Figure 2:**
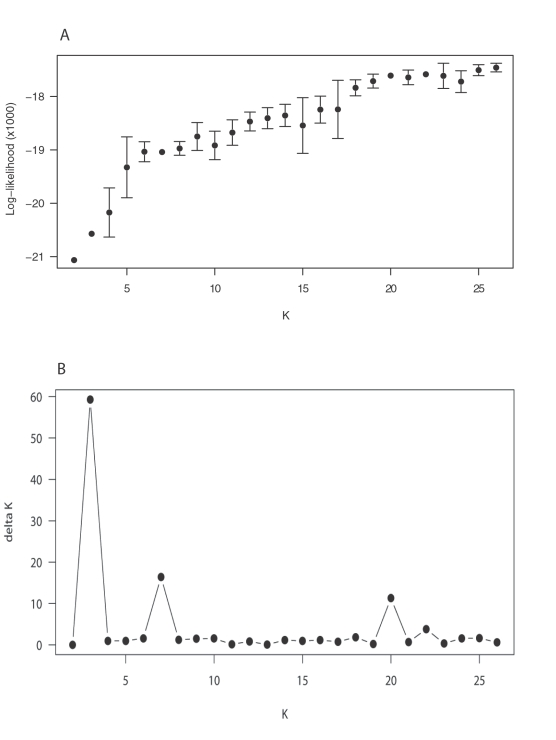
Results of the fully Bayesian model-based clustering method implemented in STRUCTURE v2.2 [21], which was used to infer the most likely number of populations (K) in the data set. For this purpose, the program was run without population information under the admixture model (individuals may have mixed ancestry) and independent allele frequencies. Length of the burn-in was 100,000 and the number of MCMC replications after the burn-in was 1,000,000. Five independent chains were run for each K from K = 2 to K = 26. A) Ln P for each K for K = 2–26, B) ΔK for each K for K = 2–26 [52].

The AMOVA (Analysis of Molecular Variance) indicates a high level of genetic structuring in this coral species (22% of the total molecular variance is partitioned among populations; based on the Infinite Allele Model, p<0.0001), suggesting that most recruitment is highly localised. This is supported by the indices of pairwise genetic differentiation (mean *F_ST_* = 0.201±0.125 SD), which are significant for all but 3 comparisons (i.e., Davies Rf 1 vs. Davies Rf 3, Yonge Reef vs. Rib Reef 10 and Rib Reef 5_2005 vs. Rib Reef 8; Table 1). The genetic composition of each of the populations is visualised in Figure 1, using the model-based clustering method implemented in STRUCTURE v2.2 [21] under the assumption that there are 20 genetic clusters (Figs. 2A, B). This analysis shows that the log probability of the data starts to plateau at a K (number of genetic clusters) of about 20 (Fig. 2A). As this coincides with one of the optima in ΔK (Fig. 2B), we have interpreted these results as 20 being the most likely number of genetic clusters in the data, although K = 2 and K = 7, the other two optima in ΔK, were also explored (discussed below but data not shown). The following patterns are revealed by the analysis based on K = 20 (Fig. 1) and these are generally supported by pairwise *F_ST_* values (Table 1): (1) Osprey Rf in the Coral Sea and the inshore Cattle Bay are genetically the most distinct, (2) Sites within a reef are in some instances as genetically distinct as sites hundreds of km apart (e.g. the Lizard Is sites), while in other cases they have *F_ST_* values not significantly different from zero (e.g., the two lagoonal sites Davies Rf 1 and 3), (3) Geographically distant sites are sometimes genetically similar (e.g., Lizard Is 2 and Agincourt Rf), (4) Populations in the Ribbon Reefs tend to be more genetically similar than populations elsewhere.

**Table 1:** Pairwise *F_ST_* values calculated using an AMOVA approach [46] in GenAlEx v6 [47].

	Osprey	Yonge	Rib 10	Rib 8	Rib 5_2005	Rib 5_2003	Lizard 1	Lizard 2	Lizard 3	Emily	Agincourt	Tongue 1	Tongue 2	Sudbury 1	Sudbury 2	Flora	Myrmidon	Cattle Bay	Davies 1	Davies 2	Davies 3	Big Broadhurst
**Osprey**																						
**Yonge**	*0.328*																					
**Rib 10**	*0.343*	0.010																				
**Rib 8**	*0.362*	*0.021*	*0.026*																			
**Rib 5_2005**	*0.370*	*0.023*	*0.025*	0.009																		
**Rib 5_2003**	*0.223*	*0.301*	*0.315*	*0.339*	*0.330*																	
**Lizard 1**	*0.369*	*0.119*	*0.094*	*0.143*	*0.130*	*0.336*																
**Lizard 2**	*0.293*	*0.265*	*0.280*	*0.297*	*0.295*	*0.196*	*0.280*															
**Lizard 3**	*0.397*	*0.090*	*0.065*	*0.094*	*0.080*	*0.374*	*0.058*	*0.310*														
**Emily**	*0.298*	*0.054*	*0.069*	*0.086*	*0.094*	*0.226*	*0.127*	*0.187*	*0.125*													
**Agincourt**	*0.292*	*0.328*	*0.340*	*0.361*	*0.358*	*0.198*	*0.349*	*0.052*	*0.384*	*0.246*												
**Tongue 1**	*0.470*	*0.079*	*0.067*	*0.092*	*0.099*	*0.413*	*0.140*	*0.342*	*0.111*	*0.079*	*0.416*											
**Tongue 2**	*0.384*	*0.076*	*0.085*	*0.119*	*0.117*	*0.326*	*0.118*	*0.262*	*0.108*	*0.050*	*0.337*	*0.059*										
**Sudbury 1**	*0.416*	*0.062*	*0.091*	*0.090*	*0.116*	*0.388*	*0.190*	*0.321*	*0.159*	*0.077*	*0.387*	*0.082*	*0.081*									
**Sudbury 2**	*0.502*	*0.136*	*0.136*	*0.156*	*0.172*	*0.457*	*0.165*	*0.367*	*0.139*	*0.113*	*0.443*	*0.038*	*0.060*	*0.072*								
**Flora**	*0.298*	*0.310*	*0.324*	*0.336*	*0.344*	*0.260*	*0.350*	*0.145*	*0.376*	*0.241*	*0.149*	*0.416*	*0.336*	*0.367*	*0.441*							
**Myrmidon**	*0.417*	*0.033*	*0.052*	*0.035*	*0.038*	*0.366*	*0.133*	*0.308*	*0.094*	*0.069*	*0.381*	*0.042*	*0.051*	*0.051*	*0.070*	*0.368*						
**Cattle Bay**	*0.416*	*0.195*	*0.211*	*0.153*	*0.215*	*0.447*	*0.323*	*0.373*	*0.299*	*0.205*	*0.419*	*0.281*	*0.282*	*0.182*	*0.301*	*0.384*	*0.216*					
**Davies 1**	*0.429*	*0.098*	*0.122*	*0.140*	*0.160*	*0.389*	*0.199*	*0.310*	*0.209*	*0.110*	*0.373*	*0.136*	*0.106*	*0.083*	*0.146*	*0.366*	*0.103*	*0.187*				
**Davies 2**	*0.292*	*0.069*	*0.096*	*0.100*	*0.110*	*0.246*	*0.152*	*0.166*	*0.160*	*0.056*	*0.219*	*0.142*	*0.076*	*0.083*	*0.152*	*0.191*	*0.083*	*0.169*	*0.064*			
**Davies 3**	*0.427*	*0.085*	*0.112*	*0.122*	*0.142*	*0.390*	*0.190*	*0.301*	*0.185*	*0.103*	*0.370*	*0.119*	*0.095*	*0.080*	*0.130*	*0.365*	*0.088*	*0.195*	0.010	*0.061*		
**Big Broadhurst**	*0.250*	*0.102*	*0.125*	*0.134*	*0.143*	*0.208*	*0.172*	*0.129*	*0.177*	*0.072*	*0.173*	*0.174*	*0.109*	*0.117*	*0.183*	*0.149*	*0.126*	*0.190*	*0.110*	*0.018*	*0.109*	

Statistical significance was assessed based on 9,999 permutations and corrected for multiple comparisons, with significant values (after FDR correction [50]) written in italics.

K = 2 is unrealistically small given the high population structure in this species based on *F_ST_* values obtained here and in previous work [15]. However, it does suggest that the following populations are admixed: Lizard Is 2, Ribbon Rf 5_2003, Emily Rf, Tongue Rf 2, Davies Rf 2 and Big Broadhurst Rf. The latter is consistent with the results of other analyses which are presented below. K = 7 shows similar patterns to K = 20, i.e., most of the Ribbon Reef sites are genetically similar; Cattle Bay is genetically very distinct; there is some affinity between Osprey and Ribbon Rf 5_2003, yet these two populations are distinct from all other populations; Lizard Is 2 is the most distinct of the three Lizard Is sites; Davies Rf 2 is genetically closer to Big Broadhurst Rf than to the two lagoonal Davies Rf sites; Agincourt Rf is similar to Lizard Is 2. Further, with K = 7 a weak latitudinal trend is distinguishable (which is not as obvious from the K = 20 analysis), but some geographically distant population pairs are genetically similar.

Approximately 2.5% of the samples (25) were found to have multi-locus genotypes that occur more than once. Eleven of those occur within sites and one sample of each pair was removed before analysis as it was assumed that clones within a collection site were produced through fragmentation. In addition, 14 multi-locus genotypes are shared between some of the sites sampled (Table 2). These allopatric clone mates were not removed from the data prior to analysis, since it concerns a relatively small number of specimens and because it is unknown which of the sites was the source. However, we performed the AMOVA with and without the allopatrically occurring repeated genotypes and obtained the same results.

**Table 2:** Locations of the 14 multi-locus genotypes found at more than one site.

Locations	Linear distance between sites (km)^2^	Probability of the multi-locus genotype being produced by sexual reproduction in each of the populations^1^
Davies 1, Davies 3	0.887	0.0096, 8.5×10^−7^
Davies 1, Davies 2	1.925	0.0010, 4.3×10^−17^
Myrmidon, Sudbury 2, Emily	346.616 (M-E), 166.161 (S-E), 184.056 (M-S)	0.0003, 0.0042, 6.3×10^−13^
Sudbury 1, Sudbury 2	10.950	0.0001, 7.4×10^−6^
Sudbury 2, Tongue 2	92.615	0.0002, 1.5×10^−6^
Sudbury 2, Tongue 2	92.615	0.0001, 0.0126
Sudbury 2, Tongue 2	92.615	0.0136, 0.0002
Sudbury 2, Tongue 2, Emily	92.615 (S-T), 166.161 (S-E), 75.663 (T-E)	0.0005, 0.0004, 1.1×10^−5^
Sudbury 2, Tongue 1	90.102	0.0002, 4.7×10^−5^
Sudbury 2, Tongue 1	90.102	0.0110, 0.0006
Sudbury 2, Emily	166.161	1.9×10^−5^, 1. 6×10^−7^
Tongue 1, Tongue 2	2.513	3.3×10^−5^, 0.0001
Tongue 1, Ribbon 8	133.781	5.4×10^−5^, 3.3×10^−8^
Tongue 1, Ribbon 10	154.086	0.0034, 0.0003

1 The probabilities of the multi-locus genotypes being produced by sexual reproduction in each of the populations was calculated in GENCLONE 2.0 [48] and shows that it is highly unlikely that any of these genotypes was produced twice or three times by sexual reproduction at different locations. Only genotypes with no missing data (13) or data for only a single locus missing (1) were used.

Geographic distances are calculated from a MGA zone 55 projection.

An exclusion test conducted in GeneClass v2.0 [22] identifies 42 individuals (∼4%) as having originated at a site other than the site these were sampled from (Table 3). While the majority of these are likely to have been sourced from unsampled populations (i.e., these individuals have extremely low probabilities of coming from any of the sampled populations), 14 of the excluded individuals can be assigned to one or more of the other sampling locations. *S. hystrix* is very common and widespread on the GBR, and many potential source populations were therefore not sampled in this study. Hence, it is possible that some immigrants have been assigned to a population they have not originated from. Given that geographically close populations are generally genetically more similar than geographically distant ones (although there are a few exceptions to this pattern), however, we are confident that the estimates of the spatial scales over which these larvae have dispersed are reasonably accurate. Some of the migrants are inferred to be sourced from nearby sites (e.g., Davies Rf versus Big Broadhurst Rf), while others appear to have been transported over distances of 10 s to 100 s of kilometres (e.g., Emily Rf versus Sudbury Rf). However, there is no assignment of migrants between the extremes of the sampling range, providing further confidence that these results provide reasonably accurate estimates of recent dispersal distances.

**Table 3:** Results of exclusion analysis conducted in GeneClass v2.0 [22].

Sampling location of the individual	self						
Osprey Rf	0.0039						
Osprey Rf	0.0028	0.2549 (Ribbon Rf 5_2003)					
Osprey Rf	0.0045						
Yonge Rf	0						
Lizard Isl 2	0.002						
Lizard Isl 2	0.0029						
Lizard Isl 3	0.0043	0.1049 (Lizard Is 1)					
Lizard Isl 3	0.0012	0.1108 (Lizard Is 2)	0.1505 (Tongue Rf 2)	**0.1667 (Emily Rf)**			
Ribbon Rf 10	0.0009						
Ribbon Rf 8	0.006	0.1164 (Emily Rf)					
Emily Rf	0.0082						
Emily Rf	0.0047						
Emily Rf	0.0038						
Emily Rf	0.0013	0.1691 (Flora Rf)	0.1193 (Lizard Is 2)	**0.7942 (Agincourt)**			
Emily Rf	0.0066						
Emily Rf	0.0043						
Agincourt Rf	0.0023						
Agincourt Rf	0.0045						
Agincourt Rf	0.0001	0.5021 (Ribbon Rf 5_2003)					
Agincourt Rf	0.0021						
Tongue Rf 2	0.0098						
Tongue Rf 2	0						
Tongue Rf 1	0						
Sudbury Rf 1	0.001	0.1683 (Tongue Rf 2)	**0.2245 (Emily Rf)**				
Sudbury Rf 2	0.0014	0.1008 (Lizard Is 2)	0.3774 (Big Broadhurst Rf)	0.1406 (Davies Rf 2)	0.1299 (Myrmidon Rf)	0.3865 (Tongue Rf 2)	**0.6609 (Emily Rf)**
Flora Rf	0.0038						
Flora Rf	0.0081						
Flora Rf	0.0013						
Myrmidon Rf	0.001						
Myrmidon Rf	0.0004						
Cattle Bay	0.0016	0.1006 (Big Broadhurst Rf)	**0.1129 (Davies Rf 2)**				
Davies Rf 1	0.0017	0.1133 (Emily Rf)					
Davies Rf 1	0.0013	0.2564 (Big Broadhurst Rf)	**0.3694 (Davies Rf 2)**	0.116 (Tongue Rf 2)			
Davies Rf 2	0.0001						
Davies Rf 2	0.0014						
Davies Rf 2	0.0042	0.5266 (Flora Rf)					
Davies Rf 2	0.0087						
Davies Rf 3	0.0005	0.1672 (Davies Rf 2)					
Davies Rf 3	0.0092	**0.2785 (Davies Rf 2)**	0.1725 (Big Broadhurst Rf)	0.2277 (Emily Rf)	0.1003 (Yonge Rf)		
Big Broadhurst Rf	0.0032						
Big Broadhurst Rf	0.0015						
Big Broadhurst Rf	0.0008						

The values shown are the probabilities that the individual originated at the sampling location (‘self’) or at any of the other sampled locations (only populations for which p>0.1 are shown). Where there is more than one potential source population, the potential source population with the highest probability is highlighted in bold face.

**Figure 3:**
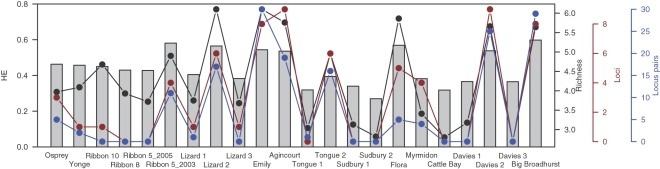
Genetic signature of recent admixture. The combination of large numbers of locus pairs in Linkage Disequilibrium (LD), heterozygote deficits at a large number of loci, and high genetic diversity (*H_E_* and allelic diversity) in some of the populations is indicative of recent admixture. HE = Expected heterozygosity, Loci = number of loci with significant heterozygote deficit, Locus pairs = number of locus pairs showing Linkage Disequilibrium, Richness = allelic richness.

Significant Linkage Disequilibrium (LD) was found in 165 out of 1,035 comparisons (15.9%), and at the following sites (numbers of locus pairs in parentheses): Osprey Rf (5), Yonge Rf (1), Ribbon Rf 5_2003 (11), Lizard Island 1 (1), Lizard Island 2 (17), Emily Rf (30), Agincourt Rf (19), Tongue Rf 1 (1), Tongue Rf 2 (16), Flora Rf (5), Myrmidon Rf (4), Davies Rf 2 (25) and Big Broadhurst Rf (29). All the sites with large numbers of LD also show significant heterozygote deficits involving 3–9 loci (Fig. 3 and Table 4). However, none of the loci exhibit significant deficits at all locations, with the highest number of significant heterozygote deficits observed for Sh2-005 (8 sites), indicating that these results are unlikely to be due to the presence of null alleles. Exact tests show significant deviations from Hardy-Weinberg Equilibrium (HWE) exist in the sites at Osprey Rf (3 loci), Yonge Rf (1 locus), Ribbon Rf 10 (1 locus), Ribbon Rf 5_2003 (4), Lizard Island 1 (1 locus), Lizard Island 2 (6 loci), Lizard Island 3 (1 locus), Emily Rf (8 loci), Agincourt Rf (9 loci), Tongue Rf 2 (6 loci), Flora Rf (5 loci), Myrmidon Rf (4 loci), Davies Rf 2 (9 loci) and Big Broadhurst Rf (8 loci), while all 10 loci are in HWE at the remaining 8 sites. Presence of extensive LD and heterozygote deficits was found mainly in locations exhibiting high levels of genetic diversity, as assessed by allelic richness and expected heterozygosity (Fig. 3).

**Table 4:** Descriptive statistics for the ten microsatellite loci at 22 sites of *S. hystrix*.

Population	Locus	Sh4-001	Sh2-002	Sh3-003	Sh3-004	Sh2-005	Sh2-006	Sh3-007	Sh3-008	Sh3-009	Sh4-010
Osprey	N	36	36	36	36	36	36	36	36	36	36
	A	3	12	2	6	2	4	4	2	4	4
	*H_E_*	0.06	0.78	0.50	0.64	0.22	0.50	0.50	0.03	0.61	0.19
	*H_O_*	0.05	0.84	0.50	0.66	0.38	0.56	0.60	0.03	0.61	0.41
	*F_IS_*	−0.007	*0.084*	0.011	0.046	0.419	0.115	*0.181*	-	0.008	*0.536*
Yonge	N	48	48	48	48	48	45	48	48	48	48
	A	4	7	3	5	3	8	4	2	3	7
	*H_E_*	0.81	0.79	0.10	0.71	0.29	0.91	0.58	0.00	0.38	0.42
	*H_O_*	0.66	0.80	0.10	0.59	0.25	0.72	0.60	0.04	0.36	0.44
	*F_IS_*	−0.213	0.024	−0.035	−0.181	−0.147	−0.255	0.033	-	*−0.025*	0.060
Ribbon 10	N	49	49	49	49	48	45	48	49	48	48
	A	6	9	5	6	3	10	4	3	5	7
	*H_E_*	0.76	0.69	0.16	0.59	0.25	0.76	0.56	0.02	0.60	0.38
	*H_O_*	0.68	0.72	0.15	0.59	0.22	0.69	0.53	0.06	0.49	0.36
	*F_IS_*	−0.101	0.051	−0.046	0.000	−0.120	−0.081	−0.056	*0.664*	−0.220	−0.019
Ribbon 8	N	50	50	50	50	50	50	50	50	50	50
	A	5	8	1	3	5	7	4	1	5	8
	*H_E_*	0.58	0.74	0.00	0.48	0.24	0.66	0.56	0.00	0.34	0.66
	*H_O_*	0.57	0.82	0.00	0.51	0.22	0.69	0.50	0.00	0.36	0.63
	*F_IS_*	−0.005	0.106	-	0.061	−0.072	0.051	−0.111	-	0.062	−0.032
Ribbon 5_2005	N	49	48	49	49	49	49	49	49	49	49
	A	4	10	5	2	3	4	4	1	4	5
	*H_E_*	0.55	0.92	0.14	0.55	0.18	0.59	0.41	0.00	0.45	0.41
	*H_O_*	0.59	0.82	0.14	0.50	0.20	0.66	0.49	0.00	0.45	0.42
	*F_IS_*	0.075	−0.107	−0.039	−0.092	0.106	0.112	0.185	-	0.017	0.048
Ribbon 5_2003	N	28	28	28	28	29	29	29	29	29	29
	A	4	5	2	7	5	10	5	3	4	5
	*H_E_*	0.61	0.57	0.21	0.71	0.45	0.66	0.10	0.10	0.66	0.69
	*H_O_*	0.66	0.70	0.44	0.73	0.74	0.75	0.36	0.10	0.66	0.69
	*F_IS_*	0.092	*0.201*	0.522	0.041	*0.411*	0.144	*0.718*	−0.024	0.019	0.013
Lizard 1	N	36	40	40	25	42	42	42	42	42	42
	A	5	7	2	2	4	6	4	1	4	5
	*H_E_*	0.50	0.57	0.03	0.60	0.21	0.60	0.45	0.00	0.50	0.55
	*H_O_*	0.54	0.55	0.02	0.47	0.24	0.48	0.61	0.00	0.56	0.56
	*F_IS_*	0.096	−0.024	-	−0.254	0.109	−0.220	*0.270*	-	0.125	0.027
Lizard 2	N	53	54	54	51	54	54	54	54	54	54
	A	6	17	4	6	12	7	6	4	5	9
	*H_E_*	0.19	0.83	0.07	0.61	0.72	0.85	0.43	0.26	0.59	0.46
	*H_O_*	0.27	0.86	0.39	0.69	0.69	0.69	0.52	0.26	0.70	0.58
	*F_IS_*	*0.320*	*0.036*	*0.814*	0.125	−0.037	*−0.229*	*0.188*	0.002	*0.167*	*0.216*
Lizard 3	N	50	48	50	49	50	50	50	50	50	50
	A	4	6	2	3	3	7	4	1	5	8
	*H_E_*	0.56	0.63	0.02	0.37	0.04	0.60	0.48	0.00	0.54	0.52
	*H_O_*	0.61	0.63	0.02	0.38	0.04	0.54	0.54	0.00	0.54	0.54
	*F_IS_*	0.084	0.018	-	*0.041*	−0.005	−0.109	0.123	-	0.012	0.053
Emily	N	50	50	50	50	50	50	50	50	50	49
	A	6	19	3	7	9	11	4	3	6	7
	*H_E_*	0.40	0.78	0.06	0.48	0.38	0.64	0.32	0.04	0.14	0.31
	*H_O_*	0.62	0.83	0.30	0.71	0.62	0.79	0.60	0.04	0.40	0.54
	*F_IS_*	*0.364*	*0.067*	*0.803*	*0.331*	*0.400*	0.196	*0.473*	−0.005	*0.658*	*0.440*
Agincourt	N	49	49	49	49	49	49	49	49	49	49
	A	7	16	3	8	6	9	5	3	8	4
	*H_E_*	0.27	0.73	0.37	0.47	0.45	0.78	0.35	0.04	0.69	0.18
	*H_O_*	0.46	0.80	0.49	0.58	0.61	0.65	0.49	0.04	0.80	0.45
	*F_IS_*	*0.429*	*0.090*	*0.260*	*0.197*	*0.269*	*−0.180*	0.296	−0.005	*0.147*	*0.596*
Tongue 1	N	49	49	49	49	50	50	50	50	50	50
	A	3	5	5	3	4	7	2	1	2	4
	*H_E_*	0.51	0.73	0.08	0.47	0.32	0.56	0.40	0.00	0.04	0.18
	H_o_	0.49	0.63	0.12	0.50	0.29	0.55	0.40	0.00	0.04	0.17
	*F_IS_*	−0.021	−0.149	0.316	0.074	−0.100	−0.016	0.018	-	−0.010	−0.059
Tongue 2	N	45	45	45	45	42	44	44	44	43	44
	A	5	14	2	6	11	6	3	3	5	7
	*H_E_*	0.31	0.44	0.04	0.38	0.21	0.66	0.34	0.05	0.14	0.36
	*H_O_*	0.57	0.54	0.08	0.46	0.43	0.69	0.58	0.04	0.19	0.35
	*F_IS_*	*0.465*	*0.189*	0.485	0.188	*0.506*	*0.062*	*0.417*	−0.006	*0.288*	−0.027
Sudbury 1	N	42	42	42	42	42	42	42	42	41	42
	A	4	4	3	2	3	7	2	1	5	5
	*H_E_*	0.74	0.40	0.05	0.60	0.24	0.55	0.38	0.00	0.12	0.38
	*H_O_*	0.65	0.52	0.05	0.50	0.28	0.56	0.34	0.00	0.12	0.38
	*F_IS_*	−0.122	0.237	−0.006	−0.180	0.175	0.031	−0.119	-	−0.028	0.009
Sudbury 2	N	47	47	47	47	47	47	47	47	47	47
	A	4	2	2	2	4	9	3	2	2	3
	*H_E_*	0.49	0.49	0.02	0.49	0.17	0.64	0.13	0.02	0.09	0.28
	*H_O_*	0.53	0.37	0.02	0.44	0.20	0.61	0.12	0.02	0.08	0.31
	*F_IS_*	0.086	−0.314	-	−0.096	0.139	−0.035	−0.047	-	−0.034	0.113
Flora	N	42	42	42	42	42	42	42	42	42	42
	A	7	14	3	7	6	5	6	4	7	6
	*H_E_*	0.74	0.86	0.45	0.48	0.48	0.24	0.36	0.33	0.33	0.38
	*H_O_*	0.75	0.86	0.66	0.70	0.54	0.29	0.33	0.51	0.54	0.51
	*F_IS_*	*0.029*	0.020	0.321	*0.330*	*0.130*	0.204	−0.058	0.351	*0.387*	*0.269*
Myrmidon	N	49	49	49	49	49	49	49	49	49	49
	A	3	9	1	2	6	5	3	2	6	4
	*H_E_*	0.67	0.84	0.00	0.47	0.14	0.71	0.41	0.02	0.06	0.33
	*H_O_*	0.54	0.69	0.00	0.47	0.26	0.69	0.52	0.02	0.16	0.35
	*F_IS_*	*−0.181*	−0.185	-	0.023	*0.447*	−0.007	*0.261*	-	*0.581*	0.160
Cattle Bay	N	47	47	47	47	47	47	47	47	47	47
	A	3	4	1	2	4	4	3	3	2	6
	*H_E_*	0.38	0.49	0.00	0.30	0.28	0.51	0.62	0.04	0.17	0.49
	*H_O_*	0.34	0.55	0.00	0.28	0.28	0.55	0.49	0.04	0.16	0.49
	*F_IS_*	−0.105	+0.122	-	−0.044	0.020	0.090	−0.261	−0.005	−0.082	0.011
Davies 1	N	42	42	42	42	42	42	42	42	42	42
	A	4	4	2	2	4	6	4	3	2	5
	*H_E_*	0.48	0.48	0.02	0.57	0.50	0.50	0.55	0.07	0.07	0.67
	*H_O_*	0.44	0.39	0.02	0.47	0.48	0.63	0.48	0.07	0.07	0.61
	*F_IS_*	−0.080	−0.200	-	−0.200	−0.024	0.212	−0.123	−0.017	−0.025	−0.080
Davies 2	N	44	44	44	44	44	44	44	44	44	44
	A	7	14	4	7	8	5	5	3	6	7
	*H_E_*	0.43	0.45	0.18	0.50	0.32	0.55	0.41	0.14	0.20	0.50
	*H_O_*	0.60	0.68	0.35	0.70	0.49	0.59	0.68	0.13	0.46	0.72
	*F_IS_*	*0.285*	*0.337*	*0.487*	*0.298*	*0.358*	*0.081*	*0.410*	−0.045	*0.562*	*0.314*
Davies 3	N	48	48	48	48	48	48	48	48	48	48
	A	3	4	2	3	4	3	2	1	3	5
	*H_E_*	0.42	0.56	0.04	0.56	0.33	0.54	0.48	0.00	0.13	0.63
	*H_O_*	0.36	0.55	0.04	0.52	0.36	0.62	0.44	0.00	0.12	0.65
	*F_IS_*	−0.154	−0.015	−0.011	−0.073	0.090	0.135	−0.085	-	−0.046	0.048
Big Broadhurst	N	51	51	51	51	51	51	51	51	51	51
	A	8	12	3	5	7	7	5	4	8	7
	*H_E_*	0.47	0.63	0.20	0.63	0.45	0.45	0.45	0.16	0.33	0.49
	*H_O_*	0.70	0.68	0.46	0.73	0.63	0.55	0.71	0.22	0.59	0.70
	*F_IS_*	*0.338*	*0.090*	*0.584*	*0.151*	*0.293*	0.182	*0.378*	0.285	*0.444*	*0.312*
Total sample (1014 individuals)	A	11	32	9	12	25	19	13	8	13	15
Allele range		130–190	128–186	82–103	138–189	104–158	105–141	104–142	210–249	148–206	210–278

N = number of samples per locus and population, A = number of alleles, *H_E_* = expected heterozygosity, *H_O_* = observed heterozygosity, *F_IS_* = inbreeding coefficient [55] (values in italics are statistically significant after FDR correction following [50]).

## Discussion

### Population structure on the GBR

This study shows a high level of genetic structuring among most GBR populations of the coral *S. hystrix*, supporting earlier findings based on allozyme analysis of the same species on the GBR [15], [16] and the fact that most larvae settle within several hours to days after release in laboratory studies [23], [24]. Recently, Underwood et al. [25] have demonstrated that most recruitment in NW Australian *S. hystrix* populations occurs within 100 m of the natal colony, a finding also supported by our data.

The extreme genetic distinctiveness of the Osprey Rf population is likely due to its geographic isolation in the Coral Sea, and that of Cattle Bay by the limited cross-shelf water exchange in the central GBR [26], [27]. Most sites in the Ribbon Reefs are genetically more similar than populations elsewhere and some exhibit pairwise *F_ST_* values not significantly different from zero, consistent with an almost continuous north-south reef matrix along the Ribbon Reefs acting as a stepping stone for coral dispersal. There are, however, exceptions to this pattern. For example, the two sites sampled at Ribbon Rf 5 are very genetically divergent (Fig. 1). This may reflect habitat differences (the mean collection depth of the two sites was 8.4 m and 3.3 m), or temporal variation (the samples were collected in different years, in this case 2003 and 2005). However, because no known major disturbance events have occurred between the two sampling time points at Ribbon Rf 5, our interpretation of the results is that they reflect independent bouts of recruitment, the Ribbon Rf 5_2003 population possibly from outside the reef. This is supported by the relative genetic similarity between some geographically distant sites (e.g., Lizard Is 2 and Agincourt Rf, see Fig. 1). While theoretically this can be the result of size homoplasy of alleles, it is unlikely to be the case here as the same most common alleles are found at all loci (data not shown). Random genetic drift could also have led to similar allele frequencies, but again, it is unlikely this would have happened at all of the 10 loci examined. The most plausible explanation therefore is that a recruitment pulse has occurred from one location to the other or from another genetically similar, but unsampled location. Higher levels of genetic differentiation within rather than between reefs is commonly observed in corals and other marine organisms [16], [28], [29], and this may reflect the spatial and temporal stochasticity of larval recruitment due to complex and temporally variable patterns of water circulation around the reef matrix [27], [30] as well as temporal variation in fecundity of marine organisms [4], [5]. To better understand the stochasticity of recruitment in reef corals, future studies should focus on the genetic characterisation of different cohorts, for example by studying distinct size classes or new recruits over the course of several recruitment cycles.

### Localised recruitment is supplemented by recent longer distance dispersal

Despite most recruitment in *S. hystrix* on the GBR being highly localised, our results suggest a considerable amount of recent exchange of both sexual and asexual larvae has occurred, and that up to ∼5.4% of the total number of colonies sampled may represent recent migrants. Fourteen multi-locus genotypes were shared between some of the sites sampled. As it is highly unlikely that coral fragments would survive transportation by water movement between reefs, these results suggest that *S. hystrix* occasionally produces asexual larvae and that those can be swept off the natal reef and settle elsewhere, in some instances more than 100 km away (Table 2). Only sexual larvae have been described for *S. hystrix*; two independent allozyme studies showed the presence of non-maternal alleles in some of the larvae from 1 colony from the central GBR [31] and 6 colonies from the southern GBR [32], indicating that the broods were sexually produced. However, reproduction is highly plastic in brooding pocilloporid corals [33], [34] and it is therefore possible that some populations produce asexual larvae. Alternatively, these results could be explained by polyp bail-out, a stress response first described in *S. hystrix* [35], where polyps detach themselves from the skeleton, disperse and re-attach to the substratum.

In addition to recent long-distance dispersal of asexually produced larvae, migration of sexually produced larvae can be inferred from the exclusion test results (Table 3). *S. hystrix* produces mature planula larvae, already containing algal endosymbionts [23], [36]. The planulae are of a range of sizes with the larger planulae having the longer survivorship, suggesting a strategy that accommodates both short and long-distance dispersal [37]. Also, we note that non-fed planulae of the related species, *Pocillopora damicornis*, are able to settle after 2 hrs in the laboratory, but ∼5% of the planulae can remain competent to settle for >103 days after release from the parental colony [38]. The presence of algal endosymbionts in the planulae may increase their survival as these are an important energy source [37]. Further, brooding corals, including *S. hystrix*, tend to show extended periods (several months) of larval release compared to broad-cast spawning corals [36], thereby increasing the probability of favourable hydrodynamic conditions for the occurrence of long-distance dispersal of larvae. Finally, it is possible that longer distance dispersal occurs through rafting of small colonies attached to floating material, such as coconuts [39].

The combination of high levels of Linkage Disequilibrium (LD), heterozygote deficits at large number of loci, and high genetic diversity in some of the populations (Fig. 3) is also indicative of recent admixture. Strong associations between physically unlinked loci are caused by the co-occurrence of alleles at different loci in the migrants and their early descendants [40]. LD among loci can be maintained for several generations and decays according to the recombination rate [41]. Dispersal of individuals among genetically distinct populations also causes heterozygote deficits due to the resulting changes in allele frequencies in the receiving population [42] and inflated genetic diversity due to the entry of new alleles into the population. This signature of recent admixture can be used to identify source and sink reefs and complements the results from the exclusion test. Recent migrants identified by the exclusion analysis occur at Osprey Rf, Emily Rf, Agincourt Rf, Tongue Rf 2, Flora Rf, Myrmidon Rf, Davies Rf 2 and Big Broadhurst Rf, consistent with a strong signature of recent admixture at these sites (Fig. 3). This suggests these reefs act as sinks, or as sinks and sources simultaneously. Near HWE has previously been found in reef lagoon populations of this species in the central GBR, but not in other habitats [15]. In the two reef areas where both lagoonal and non-lagoonal sites were sampled (Davies Rf and Lizard Is), we observed the same pattern. This suggests that lagoonal populations are generally more self-seeded than non-lagoonal populations. Based on the presence of clone mates in allopatry and the lack of a recent admixture signature, Sudbury Rf 2 is a key example of a source reef, sending migrants both north and south (Table 2). The population size of *S. hystrix* at the two Sudbury reefs was unusually large and the colonies on this reef were also large (MJHvO and AHL, personal observations). This suggests that a disproportionally large number of larvae are produced here and the likelihood of some larvae reaching other reefs and establishing themselves is relatively high.

### Conclusions

In conclusion, our study is the first in which a considerable level of recent migration over spatial scales of 10 s to 100 s of km has been shown in a brooding coral on the GBR. *S. hystrix* is extremely sensitive to heat and light stress [13], but it seems to have the potential to recover after major disturbances. While it is possible that small colony fragments survive bleaching events in crevices, sheltered from high light levels, and regrow to form large mature colonies, the results reported here indicate that some recovery is also possible through migration from external reefs.

## Materials and Methods

### Sampling of corals

Small fragments from 1,025 colonies (36–54 per site) of the coral *Seriatopora hystrix* (Scleractinia: Pocilloporidae) were collected between March 2003 and February 2005, and their genotypes at 10 microsatellite loci were determined. The samples originated from 21 collection sites on the GBR and one site in the Coral Sea (Osprey Reef) (Fig. 1), spanning ∼5 degrees or ∼500 km of latitude. Sampling occurred over a spatial scale of ∼100 m^2^ at each site. The work by Underwood et al. [25] has shown that most *S. hystrix* larvae on NW Australian reefs settle within 100 m of natal colony, suggesting that this spatial scale is appropriate for the species. At most reefs, collections were made in non-exposed/lagoonal areas, with the exception of Lizard Island and Davies Reef, where two lagoonal and one non-lagoonal site was sampled. No sampling across habitat gradients was conducted.

### Genetic characterisation of coral colonies

DNA was extracted following a slightly modified method used for the black tiger shrimp [43]. PCR amplification of the ten microsatellite loci is described in Underwood et al. [44] and was carried out in 10 µL volumes. Following PCR amplification, 5 µL were purified either by precipitation or on a Sephadex G-50 column and the products were separated on the GE Healthcare MegaBace 1000 capillary sequencer. An internal size standard (ET 400-R, GE Healthcare) was run in every sample.

### Data analysis

Chromatograms were imported into the MegaBACE Genetic Profiler Software Suite version 2 (GE Healthcare) to determine the fragment sizes (alleles) present in the samples. All automatic scoring was checked manually, and samples that yielded ambiguous or no signal were re-amplified and rerun.

Genotypic Linkage Disequilibrium (LD) and deviations from Hardy-Weinberg Equilibrium (HWE) were assessed in GENEPOP (web version 3.4) by estimation of exact p-values by the Markov chain method [45]. *F_ST_* values were calculated using an AMOVA approach [46] in GenAlEx v6 [47]. Statistical significance of pairwise *F_ST_* values was based on 9,999 permutations. The probabilities of identity by sexual reproduction were calculated using the software package GENCLONE 2.0 [48], which implements a method that takes into account the *F_IS_* estimated from each population [49] as *S. hystrix* is known to show some level of self-fertilisation [32] and deviations from HWE were observed in some of the sampled populations. Statistical significance levels for all pairwise tests were adjusted for multiple comparisons using a False Discovery Rate (FDR) method [50]. Allelic richness and its statistical significance was calculated in FSTAT [51], standardized to the smallest sample following the rarefaction method.

The fully Bayesian model-based clustering method implemented in STRUCTURE v2.2 [21] was used to infer the most likely number of genetic clusters (K) in the data set. For this purpose, the program was run without population information under the admixture model (individuals may have mixed ancestry) and independent allele frequencies. Length of the burn-in was 100,000 and the number of MCMC replications after the burn-in was 1,000,000. Five independent chains were run for each K from K = 2 to K = 26. The method of Evanno et al. [52] was used to find the most likely value of K (Figs. 2 A, B).

To identify first generation migrants, an exclusion test was conducted in GeneClass v2.0 [22]. The likelihood that an individual originated from each collection location was computed following the criterion of Rannala and Mountain [53]. This likelihood was compared with the likelihood distribution of 10,000 simulated genotypes from each sampling location [54]. To obtain a conservative estimate of recent migration, an individual was excluded from its sampling site when the probability of exclusion was greater than 99% (*P* or α≤0.01). Potential source reefs of the excluded individuals were identified based on probabilities >0.1.
